# Adrenal carcinoma

**DOI:** 10.2349/biij.2.1.e9

**Published:** 2006-01-01

**Authors:** M Muttarak, A Chotirosniramit, K Unsrisong, W Na Chiangmai

**Affiliations:** 1 Department of Radiology, Chiang Mai University, Chiang Mai, Thailand; 2 Department of Surgery, Chiang Mai University, Chiang Mai, Thailand

## HISTORY

A 67-year-old man presented with dyspepsia for 4 months. Physical examination revealed a large non-tender mass in the right upper quadrant of abdomen. Blood pressure was 120/80 mmHg. Laboratory investigations were notable for elevation of serum creatinine 2.1 mg/dL (normal 0.6-1.6 mg/dL). Urinalysis was normal. Blood leucocyte count was 6.3x103 /dL with 53% neutrophils, 38% lymphocytes, and 6% monocytes. Liver function test showed reverse albumin and globulin ratio (albumin 2.6 and globulin 5.1 g/dL). Abdominal computed tomography (CT) was performed.

## IMAGING FINDINGS

**Figure 1:**
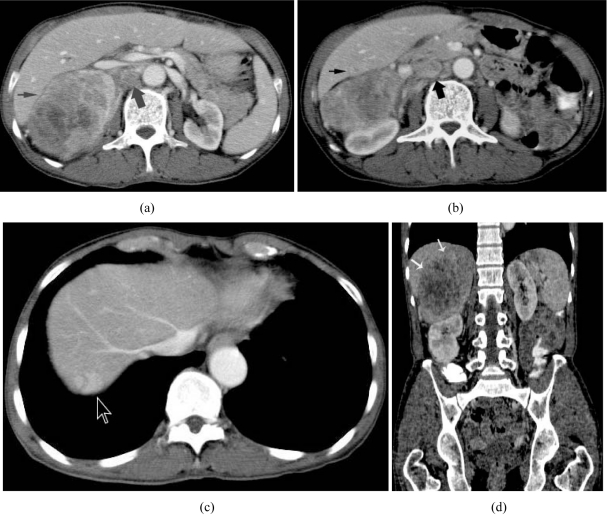
(a) Enhanced axial CT image shows a large inhomogeneous right adrenal mass (thin arrow), and an enlarged retroperitoneal lymph node (thick arrow). The inferior vena cava is patent; (b) Enhanced axial CT image obtained more distally shows a minimal inhomogeneously enhancing liver nodule (arrow), and multiple enlarged retroperitoneal lymph nodes (thick arrows); (c) Enhanced axial CT image obtained at the dome of the liver shows an enhancing liver nodule (arrow); (d) Enhanced coronal reconstructed CT image shows a large inhomogeneous right adrenal mass (arrows) with displacement of the right kidney downward.

Computed tomography of the abdomen showed a large right adrenal mass, multiple enlarged retroperitoneal nodes, and masses in the liver (Figure 1). The fat plane between the liver and the right adrenal mass was clear, indicating no direct invasion to the liver

## CLINICAL MANAGEMENT

As the imaging findings were suggestive of adrenal carcinoma with lymph node and liver metastases, biochemical assays were performed to assess a functional component of the adrenal tumour.

Urine vanillyl mandelic acid (VMA) was negative and serum cortisol level was 10.40 microgram/dL (normal 7-25 microgram/dL).

**Figure 2:**
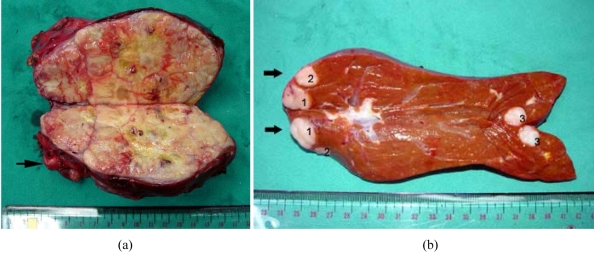
(a) Longitudinal cut section of the right adrenal gland shows solid white lobulated tissue and areas of yellow necrosis and brown haemorrhage with matted lymph nodes (arrow), which correlated to the CT findings; (b) Cut section of the right posterior segment hepatectomy shows 3 solid white nodules (1-3) with small hemorrhagic spots (arrows indicate the dome of the liver).

At surgery, the right adrenal gland was markedly enlarged with matted retroperitoneal nodes. No direct invasion to adjacent liver was noted which correlated to the CT finding. *En bloc* resection of the right adrenal gland with matted nodes, and posterior segmentectomy of the right lobe of the liver (Figure 2) were performed.

His post-operative period was complicated with subphrenic abscess. Antibiotics and percutaneous drainage of pus were administered successfully. He was subsequently discharged.

The pathological results revealed right adrenal carcinoma with lymph node and liver metastases. The inferior vena cava showed no tumour invasion.

## DISCUSSION

Adrenal carcinoma is rare, occurring with a frequency of 1 to 2 per million population per year [1]. The tumour occurs in 2 age peaks; one peak occurring at less than 5 years of age and the second peak, in the 4th and 5th decades.

Women are slightly more affected than men as shown in most studies [2-4]. Adrenal carcinoma can be classified as functional and nonfunctional; approximately 60% of cases have functional tumours.

The functional tumours are encountered more frequently in women and children, while the nonfunctional tumours tend to occur predominantly in older men [4].

The most common hormonal presentation in patients with adrenal carcinoma is Cushing syndrome, followed by Cushing syndrome with virilisation, and virilisation alone. Feminisation and Conn syndrome alone are rare presentations of this tumour [3,5].

In general, the tumour at diagnosis is large, especially those that are nonfunctional as the case described herein. The mean sizes of the carcinomas at presentation from two studies were 12 cm (range 3-30 cm) [6], and 16 cm (range 6-40 cm) [2].

CT is the imaging of choice for initial study in patients suspected of adrenal carcinomas. Abdominal CT provides information about the tumour’s resectability, renal function, and the presence of metastasis in the abdomen.

However, CT is less sensitive than magnetic resonance imaging (MRI) in evaluating tumour extension into the liver, kidney, and IVC [2,3]. On CT, adrenal carcinomas are typically large, lobulated, and inhomogeneous due to haemorrhage and necrosis. Calcifications are seen in 30% of cases [2,5].

On MRI, the tumours appear heterogeneous on both T1- and T2-weighted images owing to the presence of haemorrhage and necrosis [7].

Surgery is the most effective treatment of adrenal carcinoma. The chemotherapeutic agent often used to treat adrenal carcinoma in both primary therapy and adjuvant therapy settings is mitotane [2]. However, prognosis of patients with adrenal carcinomas is poor because the tumours are often detected at an advanced stage.
